# Emulation

**Published:** 1878-12-20

**Authors:** T. S. Powell


### *Editorial and Miscellaneous.*

☞All communications relating to the business of The Record for the years 1877 and 1878, must be addressed DR. R. C. WORD, Managing Editor Southern Medical Record, Atlanta, Ga.

☞Brief and practical communications are solicited on all subjects pertaining to medicine; also reports of cases in practice.

☞Send money by check, postal order or registered letter.

☞Write your name, post-office, county and State.

### EMULATION.

The legitimate interpretation of this word is a desire for superiority attended with an effort to obtain it by all praiseworthy means, and without any wish to repress, or oppose others.

There is a nice line of distinction between emulation and ambition. When the latter is directed toward the attainment of excellence, then it may become a synonym of the former. But it its true sense ambition is an inordinate desire of power, or eminecue, most often accompanied with illegal means to obtain the object.

When Woolsey in the midst of his fallen greatness charges Cromwell to "Fling away ambition," he saw by the dawning light of eternity that he had used a ruinous and unholy thing as a stepping stone to his own aggrandizement, regardless of good or evil to his fellow men, and he then adds the deeply portentous words, "By that sin fell the angels."

Emulation strives not only for its own good and preferment, but also for that of others. It has no petty jealousies and selfish interests, and uses no tricks of policy and malicious measures to prevent all other competors from reaching their own coveted goal. The trained racer in sweeping around the course feels but one desire, and that is to outstrip all other contestants in the race. But take two fine blooded steeds, harness them to a vehicle and start with them on a journey. How nobly they work together; how beautifully they co-operate; how the one strives to help the other, to emulate his fellow in the discharge of his duty, and accomplish the work promptly and successfully.

Emulation lifts a man cut of personal and ignoble motives, and lifts others with himself into the same pure, invigorating a mosphere. It peoples earth with beings of angelic kinship, and as by ambition the angels fell, so emulation throngs heaven's courts with cherubic and seraphic hosts that make the unceasing melodies of the eternal city.

It is a fallacy to suppose that the richest treasures of earth can be had only in exchange for gold, earth's pearls of price, paradoxical as it may seem, are to be had "without money and without price" All men, even the humblest, may emulate his competors in winning those jewels that gold cannot buy, and that are stored where thieves do not break through and steal. Every man can become a blessing to himself and his race, can be pure and good in his motives; noble, unselfish, generous and honorable in his actions; in a word, can be a peer amid the grandeur of human character, that lustrous pole star of mortal aspirations. But men sometimes say, we have no wealth, no position; we cannot influence others, or benefit them by our limited, individual efforts and resources. This is fallacious logic. True, all men cannot be leaders, comparatively few have the genius and courage to be the chief of a movement, but every subordinate can follow the leader, can strive to emulate his perseverance, his progressive activity, his hopeful spirit, and noble aspirations. The earnest, fruitful follower of a good cause, does as much for the world, and is as worthy of applause, as he who leads. However great and worthy a leader he may be he is a puissant figure of "masterly inactivity," unless he has co-cperation from his fellow men. There is no instance on record where the great Napoleon ever conceded to himself or his rare military genius the success of one of his brilliant campaigns. Whenever victory perched upon the eagles of France, his thanks were addressed to the *soldiers* of the empire

Emulation instead of being enervated by opposition, is energized, enthused, and doubly invigorated. Indeed, the blows that are struck upon it by selfish, jealous, malicious and slanderous antagonists, only serve to give it more *nerve* and forceful power, and swell its watch-word of onward and upward to louder and triumphant song. It is getting to be a popular belief that a man must do something bad before he can "succeed" as the world goes. Yes, as the world goes, ambition is an unholy thing, and to attain its unhallowed ends it must necessarily use unholy means and measures.

But our plea is not for this unclean thing; not for a power that will bring a temporary and ignoble success to the evil aspirant, and then the ultimate overthrow and ruin of himself and others—of both leader and adherents. The legitimate emulation rejoices at the success of all true and worthy men; at the full fruition of every movement or enterprise that is truly for the benefit of individuals, or mankind at large. And as a magnanimous victor never exults over a fallen foe, so the man of true emulation feels a profound sympathy and regret at the defeat of his fellow man who was nobly endeavoring to work out beneficial results for himself and the human race at the same time.

As a watchwoɪd *Excelsior* has the stigma of triteness upon it; but so long as man strives to reach after higher things, its sound like the blast of a silver trumpet cannot cease to thrill his heart as he ascends the heigths of mans noble aspirations. Higher, still higher; rings like a bugle call from the myriad tongued voices of the age, and emulation stiring within the rising and expanding soul of man, answers back the cry until it reverberates from the rivers to the ends of the earth. The thologian feels it thrilling the immortal principal within him, as he points to a christianity whose every downward step breaks one link in the golden chain that connects mans soul to blissful eternity. It makes the pure flame of love in the heart of the humanitarian burn still more brightly, till it flashes far up—ever upward, towards its natal home in the skies. Higher, still higher; cries the educator of youthful minds; higher, still higher, is written on the hypotheses of the modern philosopher, and the true scientist feels its voice cheering his labors as he reveals natures most secret arcana. And higher, still higher, should be the watchword of the physician as he explores every new field of medical science, and adapts the choicest of his gathered treasures to the relief of suffering humanity, and the edification of the ministers of medicine. It is a spirit of high and worthy emulation that must inaugerate a system of advanced excellence and superiority in our schools of education, in both literary and medical institutions.

The present systems, excellent rs they may be, are but heralds of future possibilities. Though the practice of medicine is of date immemorial, medical science is only yet in embryo. It is encouraging to know that so many of our conferes are awake to the importance of attaining the highest standard for the profession, and to the conviction that the professors of medicine must emulate a thorough, progressive, philosophical and yet, practical exposition of pathological science, etc. And if professors of medicine have this praiseworthy ambition, they should receive generous commendation and support, instead of discouragement, and the evil misrepresentation that endeavors to make them become the target for arrows from lilliputian marksmen who are compeled to remain on the lower range of the ladder, because they have neither the bright pinions of genius, nor the mighty power of intellect, to lift them above the clouds of ignorance, and they grope and travel on, with no higher aim than that of dispoiling the fame and fortune of the man of true emulation, which they can never achieve As we have endeavored to show, true emulation has no desire to oppose, or depose others. Its sole object is to reach up after the highest attainable excellence in that department towards which its energies are directed, and because is mans duty so to emulate superiorty, and to imbue others with the same spirit of laudable aspiration.

As the years roll by the path of human progress becomes more distinct, more marked, and brighter still with the light of true and sublime humanity. May we not hope that the year 1879 will notably illustrate a yet purer and broader promise for the future, and be pregnant with deeds of love, of help to tho worthy emulator; of sympathy, peace, and fraternal good will. Angel visitants, no doubt, will hover in mid air over the earth, and rejoice with humanity when a new year dawns in the universal heart of man that shall

> Bring in the truth and right.
> "Bring in the common love of good."

We again make our grateful acknowledgements to our patrons, and hope they will renew their subscriptions, and continue to aid us by their courtesy, kindness, and practical suggestions. We have uniformly received cordial commendation and encouragement during the past year, and we hope to repay the confidence and support extended us by making the RECORD still more worthy of patronage. The next volume will contain additional reading matter, with new and improved features; in a word, we shall endeavor to have the RECORD emulate all that is progressive, best, and highest in medical science and medical journalism.

Among the notable events in the past year, none has been more prominent than the yellow fever scourge that has desolated some of the fairest portions of our country; and to our bretheren who attacked the monster in its stronghold, is due the thanks and eulogistic memorials of the profession. We sincerely congratulate those who escaped the danger, and for them who fell a willing sacrifice, we write this noblest of all epitaphs, "No man hath greater love than this, that he lay down his life for another." The names of both these living and dead heroes will be *memoria in eterna* in the hearts of their grateful countrymen.

To our patrons and readers we cordially extend the compliments of the season. May the new year bring to them a "surcease of sorrows" and the boon of earth's best and truest joys!

